# Prevalence of human and non-human primate *Plasmodium* parasites in anopheline mosquitoes: a cross-sectional epidemiological study in Southern Vietnam

**DOI:** 10.1186/s41182-019-0139-8

**Published:** 2019-01-23

**Authors:** Vu Duc Chinh, Gaku Masuda, Vu Viet Hung, Hidekazu Takagi, Satoru Kawai, Takeshi Annoura, Yoshimasa Maeno

**Affiliations:** 1 Department of Entomology, National Institute of Malariology, Parasitology and Entomology, Hanoi, Vietnam; 2 Center for Southeast Asian Studies, Kyoto University, Kyoto, Kyoto Japan; 3 Department of Microbiology and Immunology, Aichi Medical University School of Medicine, Nagakute, Aichi Japan; 4 Laboratory of Tropical Medicine and Parasitology, Dokkyo Medical University, Mibu, Tochigi Japan; 5 Department of Parasitology, National Institute of Infectious Diseases, Tokyo, Japan; 6 Department of Virology and Parasitology, Fujita Health University School of Medicine, 1-98 Kutsukake, Toyoake, Aichi 470-1192 Japan

**Keywords:** *Anopheles* mosquito, Malaria transmission, Nested PCR, *Plasmodium* spp., Zoonotic malaria

## Abstract

**Background:**

Human malaria is a major threat in rural communities of central Vietnam. *Anopheles dirus* and *Anopheles minimus* species are critical malaria vectors in Vietnam, which transmit *Plasmodium* parasites. However, the entomological aspects of malaria transmission in some of the central provinces of Vietnam remain unexplored. Hence, a cross-sectional entomological survey was carried out to identify the malaria vector species and the transmission of *Plasmodium* parasites in seven endemic provinces of Vietnam.

**Methods:**

Mosquitoes were collected from seven provinces, Gia Lai, Khanh Hoa, Phu Yen, Ninh Thuan, Binh Thuan, Dong Nai, and Binh Phuoc. The collection was conducted for four to eight consecutive nights using three established methods, indoor and outdoor human landing catches and light trap method. Nested-PCR analysis was performed to detect the *Plasmodium* species in the separated thorax and the abdomen of the individual mosquitoes.

**Results:**

A total of 2278 mosquitoes belonging to one of the four species of anopheline mosquitoes, *An. dirus*, *An. maculatus*, *An. aconitus*, and *An. minimus* were collected. Among the collected mosquitoes, 1398 were analysed using nested-PCR, of which, 40 mosquitoes were positive for *Plasmodium* parasites. Most of these parasites were detected in the samples from the thorax region, followed by the abdominal portion. The parasites were detected in both the thorax and abdomen of *An. dirus*. Seven species of *Plasmodium* parasites were detected during the analysis, of which, *Plasmodium inui* was the most common species, followed by *Plasmodium falciparum*, *Plasmodium vivax*, *Plasmodium cynomolgi*, *Plasmodium coatneyi*, *Plasmodium knowlesi*, and *Plasmodium fieldi*. Out of the 49 positive samples, 12 showed mixed infections. Co-infection of *P. inui* with human and other non-human primate *Plasmodium* species was common.

**Conclusions:**

This study demonstrated the presence of human and non-human primate *Plasmodium* infection in *An. dirus*, a predominant malarial vector. Further, we showed that *An. maculatus* and *An. minimus* species also take part in malarial transmission. This might potentially lead to an alarming situation conducive for the emergence of novel zoonotic malaria.

**Electronic supplementary material:**

The online version of this article (10.1186/s41182-019-0139-8) contains supplementary material, which is available to authorized users.

## Background

Malaria is the most important infection caused by protozoans in humans. “Forest malaria” is a term frequently used to describe malaria prevalence in Southeast Asia. Transmission of *Plasmodium* parasites have been extensively found in the forest areas of southern and central Vietnam [1, 2]. *Plasmodium knowlesi*, a primate malarial parasite, has been detected in both human and *Anopheles dirus* in the central region of Vietnam [1, 3–6]. Furthermore, *An. dirus* from the Khanh Phu Commune, Khanh Vinh District, Khanh Hoa Province, was positive for both human and non-human primate *Plasmodium* parasites, of which, *Plasmodium vivax* was the most common species, followed by *P. knowlesi*, *P. inui*, *P. cynomolgi*, *P. coatneyi*, and *P. falciparum* [3, 4]. In few provinces of Southern Vietnam, residents have difficulty accessing malaria prevention services, especially the vector control measures, because of the local cultivation habits associated with sleeping in the forest and farm huts. These human behaviours and several environmental factors contribute to the risk of sylvatic transmission of *Plasmodium* parasites leading to zoonotic malarial infection among these people in Southern Vietnam [1, 6]. Since malaria control is quite unsuccessful in remote areas such as mountainous and forest areas, malaria continues to be an important health problem [1, 2, 7, 8].

The present study focuses on the determination of the various malaria vector species and their role in malaria transmission. We also demonstrated the prevalence of *Plasmodium* parasite infection in the vector species in seven provinces, Gia Lai, Khanh Hoa, Phu Yen, Ninh Thuan, Binh Thuan, Dong Nai, and Binh Phuoc of Vietnam. Hence, a detailed analysis of the mosquitoes and *Plasmodium* parasites infected vectors in these areas are necessary to assess the risk of potential zoonotic infection with non-human primate parasites in Southern Vietnam.

## Materials and methods

**Fig. 1:**
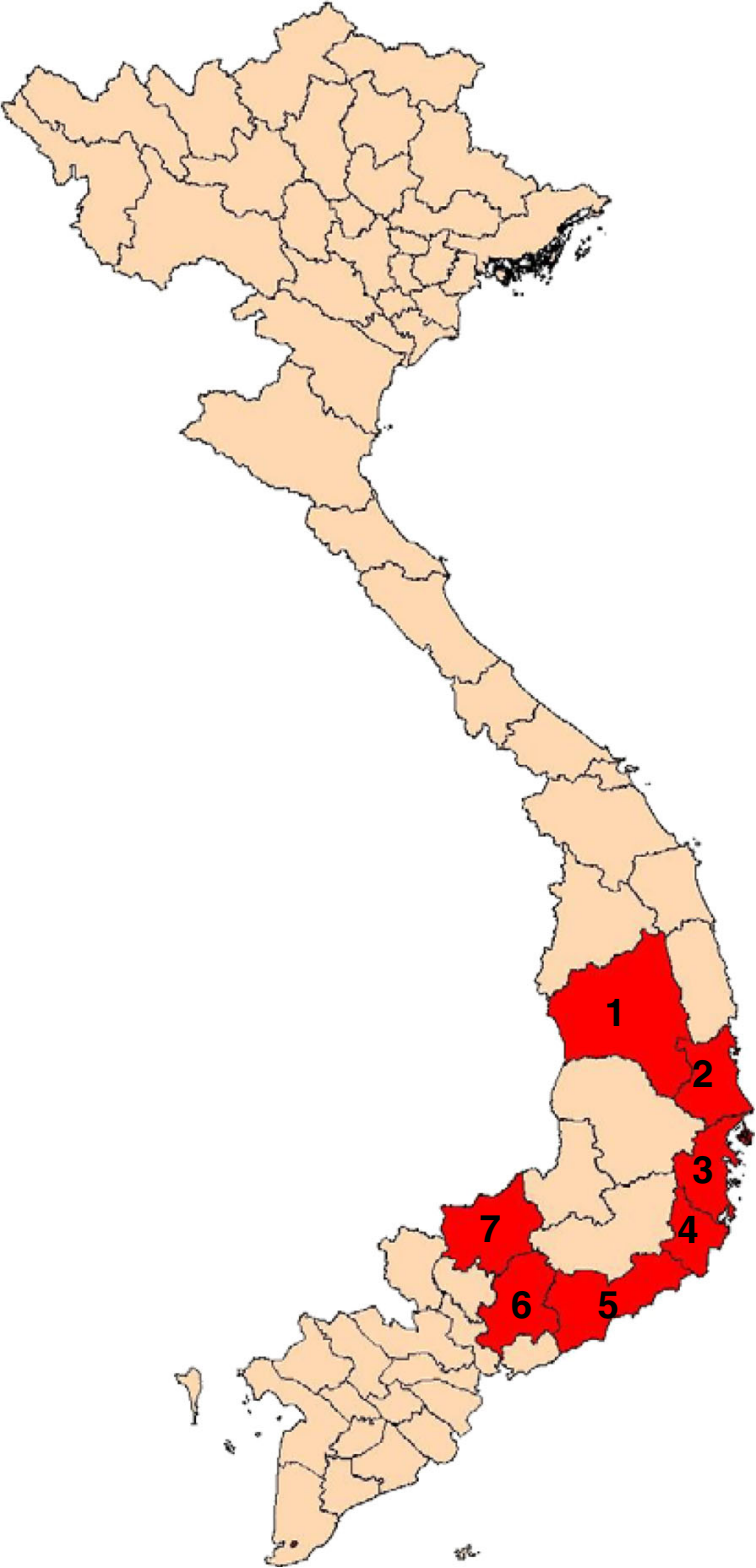
A geographic map of the study areas. 1, Gia Lai Province; 2, Phu Yen Province; 3, Khanh Hoa Province; 4, Ninh Thuan Province; 5, Binh Thuan Province; 6, Dong Nai Province; 7, Binh Phuoc Province

Mosquitoes were collected in 10 communes from 7 provinces (Fig. 1); Chu Rcam (13°30′ N; 108°60′ E) and Ia R Sai (13°33′ N; 108°62′ E) communes, Krong Pa District, Gia Lai Province; Ea Charang (13^o^3′ N; 108^o^52′ E) Commune, Son Hoa District, Phu Yen Province; Son Thai (12°24′ N; 108°81′ E) and Khanh Thuong (12°28′ N; 108°77′ E) communes, Khanh Vinh District, Khanh Hoa Province; Phuoc Binh (11^o^57′ N;108^o^45′ E) Commune, Bac Ai District, Ninh Thuan Province; Phan Tien (11^o^18′ N; 108^o^10′ E) Commune, Bac Binh District, Binh Thuan Province; Hieu Liem (11^o^10′52′′ N; 106^o^57′ E) Commune, Vinh Cưu District, Dong Nai Province; and Bu Gia Map Commune (11^o^56′ N; 106 ^o^59′ E), Binh Phuoc Province; at the beginning of the rainy season (May) and the beginning of the dry season (December) between 2005 and 2018. However, as rainy and dry seasons commence early in Gia Lai, Dong Nai, and Binh Phuoc provinces, mosquito collection was carried out a month prior. Mosquitoes were collected at the hamlet and farm hut sites within the study area. The hamlet sites are small settlements away from the forest, with people living in one-storied, brick or wooden houses and using mosquito nets. The farm hut sites are located at the forest fringe, with simple huts located at a distance from each other and no electricity. Some people at such sites do not use mosquito nets. Moreover, these areas were previously reported to be hyper- to holo-endemic for malarial infections.

Mosquito collection was conducted for four to eight consecutive nights using three different collection methods—indoor human landing catches (IHLC), outdoor human landing catches (OHLC), and indoor light traps (ILT). IHLC and OHLC methods were conducted in the field from 18:00 to 24:00 h. Mosquitoes were morphologically distinguished in the field using a standardised method to identify anopheline mosquitoes in Southeast Asia [9]. Mosquitoes were stored in small tubes over silica gel. The tubes were labelled individually by collected site, collected time, and the collection methods for subsequent analyses. This study was reviewed and approved by the research ethics committee of the National Institute of Malariology, Parasitology, and Entomology, Ministry of Health, Vietnam (366/QD118-VSR) and by the ethics committee of Fujita Health University, Japan (HM17-050). People involved in the collection process were informed about the objectives, processes, and procedures of this study. Oral informed consent was obtained from them, and they were regularly screened for malaria for 1 month and in case of infection, they were promptly treated with artemisinin-based combination therapy (ACT).

DNA was extracted from the thoracic and abdominal portions of the mosquitoes, and subsequent PCR analysis was carried out as previously described elsewhere [3]. The dissection instruments were sterilised using alcohol and flame to prevent contamination. Genomic DNA was extracted using REDExtract-N-Amp Tissue PCR Kit (Sigma-Aldrich, MO, USA). Nested-PCR assays to specifically identify human and non-human primate *Plasmodium* species were performed as described previously [10, 11]. The genus-specific primers, rPLU-1/rPLU-5, were used in the primary amplification (nest 1) and performed as described earlier. Detection of species-specific *18S rRNA* (nest 2) was performed as previously described [10, 11]. For nest 2, 2 μL of 50× nest 1 amplification product (diluted with PCR grade water) was used as the template. The PCR products were separated by electrophoresis on 1.5% agarose gels and visualised after staining with ethidium bromide. The DNA bands were analysed using Lane and Spot Analyzer software (Atto, Tokyo, Japan). To confirm the *P. knowlesi* infection, circumsporozoite protein (CSP) gene was detected as previously described [12]. Primer sequences for *18S rRNA* of human malaria parasites and primers to amplify CSP gene of *P. knowlesi* were as previously described [10–12]. A sample was considered positive for *P. knowlesi* only if both the assays (Kn1f/Kn3r and PkCSP) give positive results.

The *18S rRNA* PCR amplicons of the non-human primate *Plasmodium* species were subjected to PCR clean-up using Wizard SV Gel and PCR Clean-up System (Promega, Tokyo, Japan) according to the manufacturer’s instructions. These products were then used for sequencing analysis with the BigDye Terminator v3.1 Cycle Sequencing Premix Kit (Applied Biosystems, Inc. Tokyo, Japan). The sequencing products were run on an ABI/Hitachi 3130 × 1 Genetic Analyzer (ABI), and the resultant nucleotide sequences were analysed using software Genetyx version 11 (Genetyx Corporation, Tokyo, Japan).

## Results and discussion

In the present study, we focused on four *Anopheles* species—*An. dirus*, *An. minimus*, *An. maculatus*, and *An. aconitus—*as they are regarded as the primary and secondary malaria vectors in Vietnam. In total, 2278 mosquitoes belonging to the four *Anopheles* species were collected during the survey in the study provinces (Table 1). Number of collected mosquitoes in Khanh Hoa, Gia Lai, Phu Yen, Ninh Thuan, Binh Thuan, Dong Nai, and Binh Phuoc provinces were 332, 433, 63, 8, 437, 32, and 573, respectively. Among the four collected *Anopheles* species, *An*. *dirus* was the most common species (68%), followed by *An*. *maculatus* (21%), *An. aconitus* (8%), and *An. minimus* (3%) (Table 1). *An. dirus* and *An. minimus*, the critical vectors for *Plasmodium parasites*, were mainly collected by OHLC method (73%), followed by ILT (19%) and IHLC methods (8%) (Additional file 1). These results are because of the difference in environmental factors such as houses and differences in human behaviour depending on the collection site. [13–15]. Moreover, such differences can also be because of differences in biting behaviour of malaria vector mosquitoes [14–17].

**Table 1:** Sample collection data by indoor and outdoor human landing catches method and indoor light traps method in the study sites

		No. of mosquitoes collected				
Province	Sites	*An. dirus*	*An. maculatus*	*An. minimus*	*An. aconitus*	Total
Khanh Hoa	Khanh Thuong	51	5	2	2	60
	Son Thai	262	8	2	0	272
Gia Lai	Chur R Cam	32	116	52	187	387
	Ia Rsai	37	8	1	0	46
Phu Yen	Son Hoi	0	165	7	0	172
	Ea Charang	141	149	0	1	291
Nimh Thuan	Phuoc Binh	8	0	0	0	8
Binh Thuan	Phan Tien	419	18	0	0	437
Dong Nai	Hieu Liem	32	0	0	0	32
Binh Phuoc	Bu Gia Map	561	6	6	0	573
Total		1543	475	70	190	2278

In this study, 1386 mosquitoes randomly selected among the collected mosquitoes were subjected to nested PCR analysis for the detection of *Plasmodium* parasites (Table 2). The analysis indicated that 40 mosquitoes were positive for *Plasmodium* DNA. Among the 40 PCR-positive mosquitoes, the samples positive for *Plasmodium* DNA in the thorax, abdomen, and both regions were 20, 14, and 6, respectively (Additional file 2). From the sample collection data, we observed that 12/288 were positive in Khanh Hoa Province, 1/264 in Gia Lai Province, 3/133 in Phu Yen Province, 1/8 in Ninh Thuan Province, 1/32 in Dong Nai Province, and 23/573 in Binh Phuoc Province. No samples were tested positive in Binh Thuan Province (0/100) (Table 2). These PCR-positive samples were detected in *An. dirus* (37/1097), *An. minimus* (2/68), and *An. maculatus* (1/137) but not in *An. aconitus* (0/96). These mosquitoes were collected in the fields by ILT and OHLC methods but not by IHLC method (Additional file 3). These results were suggested to be dependent on differences in environmental factors, human behaviour, and mosquito behaviour [13–17].

**Table 2:** PCR analyses to identify the *Plasmodium* species in *An. dirus*, *An. maculatus*, *An. minimus* and *An. aconitus* from the study sites

		*An. dirus*		*An. maculatus*		*An. minimus*		*An. aconitus*		Total	
Province	Sites	No. of samples analysed	No. of positive	No. of samples analysed	No. of positive	No. of samples analysed	No. of positive	No. of samples analysed	No. of positive	No. of samples analysed	No. of positive
Khanh Hoa	Khanh Thuong	50	3	5	0	2	0	2	0	59	3
	Son Thai	226	9	1	0	2	0	0	0	229	9
Gia Lai	Chur R Cam	32	0	46	0	50	1	93	0	221	1
	Ia Rsai	37	0	5	0	1	0	0	0	43	0
Phu Yen	Son Hoi	0	0	65	0	7	0	0	0	72	0
	Ea Charang	51	2	9	1	0	0	1	0	61	3
Nimh Thuan	Phuoc Binh	8	1	0	0	0	0	0	0	8	1
Binh Thuan	Phan Tien	100	0	0	0	0	0	0	0	100	0
Dong Nai	Hieu Liem	32	1	0	0	0	0	0	0	32	1
Binh Phuoc	Bu Gia Map	561	21	6	0	6	1	0	0	573	22
Total		1097	37 (3.4%)	137	1 (0.7%)	68	2 (2.9%)	96	0 (0%)	1398	40 (2.9%)

Using species-specific PCR analysis, seven *Plasmodium* species were detected from 49 mosquito samples consisting of 28 samples of the thorax and 21 samples of the abdomen. Among the 49 PCR-positive samples, *P. inui* was the most common species (19/49), followed by *P. falciparum* (18/49), *P. vivax* (14/49), *P. cynomolgi* (9/49), *P. coatneyi* (3/49), *P. fieldi* (1/49), and *P. knowlesi* (1/49) (Table 3). Among these samples, mono-infection of six *Plasmodium* species was detected. Number of samples with mono-infection positive for *P. falciparum*, *P. vivax*, *P. inui*, *P. cynomolgi*, *P. coatneyi*, and *P. fieldi* were 14, 5, 10, 6, 1, and 1, respectively. Mixed infections with two or three *Plasmodium* species were detected in 12 samples. We observed that the co-infection of *P. inui* or *P. vivax* with human and non-human primate *Plasmodium* species was the most common, followed by *P. falciparum* co-infection with human and non-human primate *Plasmodium* parasites (Table 3). In addition, we also observed that there was no significant change in the *Plasmodium* infection rate with respect to its presence in the thorax or abdomen of the vector.

**Table 3:** Summary of *Plasmodium* spp. infection in the thorax and abdomen in the collected mosquitoes

		No. of mosquitoes infected		
Infection	*Plasmodium* spp.	Total	Thorax	Abdomen
Single (37)	Pf	14	7	7
	Pv	5	3	2
	Pin	10	4	6
	Pcy	6	5	1
	Pct	1	0	1
	Pfil	1	0	1
Double (8)	Pf, Pv	2	0	2
	Pf, Pk	1	1	0
	Pf, Pin	1	0	1
	Pv, Pin	3	3	0
	Pin, Pct	1	1	0
Triple (4)	Pv, Pin, Pcy	3	3	0
	Pv, Pin, Pct	1	1	0
No. of PCR-positive		49	28	21
No. of PCR-negative		1349	1370	1377
No. of samples analysed		1398	1398	1398

*Pf*, *Plasmodium falciparum*; *Pv*, *P. vivax*; *Pin*, *P. inui*; *Pcy*, *P. cynomolgi*; *Pct*, *P. coatneyi*; *Pfil*, *P. fieldi*; *Pk*, *P. knowlesi*

From previous literature, it is known that at least 30 of about 450 *Anopheles* species are known vectors of human and non-human primate *Plasmodium* parasites [3, 18]. In the Southeast Asian countries, *An. dirus*, *An. minimus*, *An. maculatus*, *An. epiroticus*, *An. jeyporiensis*, *An. aconitus*, *An. sinensis*, and *An. sundaicus* are the common vectors of *Plasmodium* parasites [2, 13, 19–21]. In the present study, four *Anopheles* species were collected from 10 different sites in Vietnam using 3 established collection methods. Previous studies demonstrated *An. dirus* and *An. minimus* to be the primary malaria vectors. *An. dirus* is considered the most dangerous malaria vector in Vietnam at present. *An. maculatus* and *An. aconitus* are the known secondary vectors for malarial infections [2–5, 13]. Among the mosquitoes collected in this study, *An. dirus* was the dominant vector species, whereas, *An. minimus* was present in small number. Previous long-term malaria project in Khanh Phu Commune near the Son Thai and Khanh Thuong communes of Khanh Vinh District, Khanh Hoa Province, reported that *An. minimus* was the major vector mosquito. But recently, *An. dirus* has emerged to be the dominant species [13]. Changes in the dominant species of the vector mosquitoes could be attributed to the environmental changes, modernisation, and the mosquito response after a prolonged exposure to insecticides used in malarial control [13, 15, 22].

In this study, two species of human parasites, *P. falciparum* and *P. vivax* and five species of non-human primate parasites, *P. inui*, *P. cynomolgi*, *P. coatneyi*, *P. knowlesi*, and *P. fieldi* were identified from the thorax and abdomen of the vector mosquitoes. Approximately, one-fourth of the vector mosquitoes were infected with two or three *Plasmodium* species, often in combination of human and non-human primate parasites. Similar findings were reported in a previous study in Khanh Phu Commune [3]. All these parasites are transmitted by anopheline mosquito vectors, which not only naturally infect macaques but also bite humans to cause malaria. These results indicated that these areas are at the risk of infection with both human and non-human primate *Plasmodium* parasites. These results were found to be applicable not only in a limited area but also in a wide region, further emphasising the potential risks involved in the outbreak of massive zoonotic malarial attack.

## Conclusions

The dominant species transmitting the *Plasmodium* parasites in the provinces of Vietnam chosen for this study was *An. dirus*. By PCR analysis, seven species of *Plasmodium* parasites, *P. inui*, *P. falciparum*, *P. vivax*, *P. cynomolgi*, *P. coatneyi*, *P. knowlesi*, and *P. fieldi* were detected in the vectors. Twelve out of 49 PCR-positive samples showed mixed species infection, most of which were a co-infection of *P. inui* with *P. vivax* or non-human primate *Plasmodium* species. These results suggest that humans are at a risk for infection with both human and non-human primate *Plasmodium* parasites, leading to a situation conducive for the emergence of novel zoonotic malaria in Southern Vietnam.

## Additional files

Additional file 1: Number of mosquitoes collected using different collection methods; outdoor human landing catches (OHLC), indoor human landing catches (IHLC), and indoor light traps (ILT). (DOCX 16 kb)

Additional file 2: Results of the detected *Plasmodium* spp. infection in the thorax and abdomen of the collected mosquitoes from the study sites. (DOCX 17 kb)

Additional file 3: Results of the detected *Plasmodium* spp. infection by collection method, outdoor human landing catches (OHLC), indoor human landing catches (IHLC), and indoor light traps (ILT). (DOCX 18 kb)
