# Supplementary material for: Prevalence of human and non-human primate Plasmodium parasites in anopheline mosquitoes: a cross-sectional epidemiological study in Southern Vietnam
Source: Trop Med Health. 2019 Jan 23;47:9. doi: 10.1186/s41182-019-0139-8 (PMC6343293; doi:10.1186/s41182-019-0139-8)
Supplement: Supplementary file 1 — Number of mosquitoes collected using different collection methods; outdoor human landing catches (OHLC), indoor human landing catches (IHLC), and indoor light traps (ILT). (DOCX 16 kb) [file 41182_2019_139_MOESM1_ESM.docx]

**Additional file 1** Number of mosquitoes collected using different collection methods; outdoor human landing catches (OHLC), indoor human landing catches (IHLC) and indoor light traps (ILT).

| SPECIES | Method | | | TOTAL |
| --- | --- | --- | --- | --- |
|  | OHLC | IHLC | ILT |  |
| *An. aconitus* | 171 | 7 | 12 | 190 |
| *An. dirus* | 1083 | 121 | 339 | 1543 |
| *An. maculatus* | 361 | 41 | 73 | 475 |
| *An. minimus* | 42 | 7 | 21 | 70 |
| Total | 1657 | 176 | 445 | 2278 |
