# Supplementary material for: Prevalence of human and non-human primate Plasmodium parasites in anopheline mosquitoes: a cross-sectional epidemiological study in Southern Vietnam
Source: Trop Med Health. 2019 Jan 23;47:9. doi: 10.1186/s41182-019-0139-8 (PMC6343293; doi:10.1186/s41182-019-0139-8)
Supplement: Supplementary file 2 — Results of the detected Plasmodium spp. infection in the thorax and abdomen of the collected mosquitoes from the study sites. (DOCX 17 kb) [file 41182_2019_139_MOESM2_ESM.docx]

**Additional file 2**. Results of the detected *Plasmodium* spp. infection in the thorax and abdomen of the collected mosquitoes from the study sites.

|  |  |  |  |  | Thorax | Abdomen | Both |
| --- | --- | --- | --- | --- | --- | --- | --- |
| Province | Sites | | No. examined | No. infected | No. infected | No. infected | No. infected |
| Khanh Hoa | Khanh Thuong | | 59 | 3 | 1 | 1 | 1 |
|  | Son Thai | | 229 | 9 | 2 | 6 | 1 |
| Gia Lai | Chur R Cam | | 221 | 1 | 1 | 0 | 0 |
|  | Ia Rsai | | 43 | 0 | 0 | 0 | 0 |
| Phu Yen | Son Hoi | | 72 | 0 | 0 | 0 | 0 |
|  | Ea Charang | | 61 | 3 | 3 | 0 | 0 |
| Nimh Thuan | Phuoc Binh | | 8 | 1 | 1 | 0 | 0 |
| Binh Thuan | Phan Tien | | 100 | 0 | 0 | 0 | 0 |
| Dong Nai | Hieu Liem | | 32 | 1 | 1 | 0 | 0 |
| Binh Phuoc | Bu Gia Map | | 573 | 22 | 11 | 7 | 4 |
| Total |  |  | 1398 | 40 (2.9%) | 20 | 14 | 6 |
