# Supplementary material for: Prevalence of human and non-human primate Plasmodium parasites in anopheline mosquitoes: a cross-sectional epidemiological study in Southern Vietnam
Source: Trop Med Health. 2019 Jan 23;47:9. doi: 10.1186/s41182-019-0139-8 (PMC6343293; doi:10.1186/s41182-019-0139-8)
Supplement: Supplementary file 3 — Results of the detected Plasmodium spp. infection by collection method, outdoor human landing catches (OHLC), indoor human landing catches (IHLC), and indoor light traps (ILT). (DOCX 18 kb) [file 41182_2019_139_MOESM3_ESM.docx]

**Additional file 3** Results of the detected *Plasmodium* spp. infection by collection method, outdoor human landing catches (OHLC), indoor human landing catches (IHLC) and indoor light traps (ILT).

| SPECIES | OHLC | | IHLC | | ILT | | TOTAL | |
| --- | --- | --- | --- | --- | --- | --- | --- | --- |
|  | No. examined | PCR (+) | No. examined | PCR (+) | No. examined | PCR (+) | No. examined | PCR (+) |
| *An. aconitus* | 77 | 0 | 7 | 0 | 12 | 0 | 96 | 0 |
| *An. dirus* | 830 | 24 | 52 | 5 | 215 | 8 | 1097 | 37 |
| *An. maculatus* | 62 | 1 | 30 | 0 | 45 | 0 | 137 | 1 |
| *An. minimus* | 40 | 1 | 7 | 1 | 21 | 0 | 68 | 2 |
| Total | 1009 | 26 | 96 | 6 | 293 | 8 | 1398 | 40 |
